# Transient Neonatal Diabetes Mellitus followed by recurrent asymptomatic hypoglycaemia: a case report

**DOI:** 10.1186/s12887-015-0512-7

**Published:** 2015-12-02

**Authors:** Archana Priyadarshi, Charles F. Verge, Leah Vandervliet, Deborah JG Mackay, Srinivas Bolisetty

**Affiliations:** Royal Hospital for Women, Randwick, Sydney Australia; Sydney Children’s Hospital, Randwick, Sydney Australia; School of Women’s and Children’s Health, University of New South Wales, Sydney, Australia; Faculty of Medicine, University of Southampton, Southampton, UK

**Keywords:** Transient Neonatal Diabetes Mellitus, 6q24 gene defect, Hypoglycaemia

## Abstract

**Background:**

Transient Neonatal Diabetes Mellitus is the commonest cause of diabetes presenting in the first week of life. Majority of infants recover by 3 months of age but are predisposed to developing type 2 diabetes later on in life. This condition is usually due to genetic aberrations at the 6q24 gene locus, and can be sporadic or inherited. This disorder has three phases: neonatal diabetes, apparent remission, relapse of diabetes.

**Case Presentation:**

Our case, a neonate presented with low birth weight and growth retardation along with the metabolic profile consistent with transient diabetes mellitus at birth. We report a novel clinical observation of recurrent asymptomatic hypoglycaemia detected on pre-feed blood glucose level monitoring in our case with transient neonatal diabetes mellitus at 6 weeks of age, 4 weeks after the remission of diabetes mellitus.

**Conclusion:**

This case demonstrates that neonates in remission following transient diabetes mellitus can present with recurrent asymptomatic hypoglycaemia without any other obvious congenital malformations seen. This asymptomatic hypoglycaemia may persist for weeks and may be missed if pre-feed blood glucose level monitoring is not done in these infants. Also, these infants may require an aggressive enteral feeding regimen with high glucose delivery rate to maintain normoglycemia.

## Background

Transient Neonatal Diabetes Mellitus (TNDM) is a rare genetic form of diabetes with incidence between 1:400,000 and 1:500,000 [1]. TNDM presents in the neonatal period requiring insulin treatment, remits by 18 months of age (median 3 months) and may relapse in later childhood or adolesence [2, 3]. This condition can be sporadic or inherited, with 70 % of the cases due to defects at TNDM locus on chromosome 6q24 [1, 2].

We report a case of TNDM due to a de novo 6q24 duplication arising on paternally derived chromosome who developed recurrent asymptomatic hypoglycaemia during the remission phase.

## Case presentation

Our case was a male infant, conceived by in-vitro fertilisation with donor sperm, born at 37 weeks gestation with a birth weight of 2125 gms(5^th^ percentile), length 44.5cms (5^th^ percentile) and head circumference 30.5cms (1^st^ percentile). He was delivered via caesarean section due to intrauterine growth restriction (IUGR) suspected in the last trimester of pregnancy. There was no maternal history of gestational diabetes or hypertension and maternal infection serology was negative. The infant was noted to have mild pitting oedema of lower limbs, extending up to the umbilicus, which spontaneously resolved by day 3 of life. He also had mild macroglossia. There was no family history of diabetes apart from the history that his maternal grandmother developed Type 2 Diabetes in her 80s.

Formula feeds (upon parental request) were commenced soon after birth at 60 ml/kg/day and were given as third hourly bolus feeds. At 10 h of age, the infant was detected to have hyperglycaemia with blood glucose level (BGL) of 18 mmol/L on routine pre-feed monitoring performed for intrauterine growth restriction, as per unit policy. Subsequent pre-feed BGL remained consistently high (8.0–15.6 mmol/L) over the next 12 h with glycosuria but no ketonuria. This was managed with diluted formula feeds (¼ strength) given as hourly bolus feeds, with rapid resolution of hyperglycaemia and no insulin treatment was required. On day 3 of life full strength formula feeds were re-introduced gradually increasing to 150 ml/kg/day as 3^rd^ hourly sucking feeds. However, on day 7 of life, there was a relapse of hyperglycaemia (BGL 19 mmol/L) associated with poor feeding, weight loss of 14.8 % since birth, and non-bilious vomiting. This was managed with intravenous insulin infusion 0.02 U/kg/hr with continous tube feeds of standard term infant formula (20 kcal/30 mL). Insulin levels measured at the time of hyperglycemia (BGL 11.3 mmol/L) and before insulin treatment were undetectable (<2 mU/L), with low C peptide level of 0.1 nmol/L, beta-hydroxybutyrate 0.09 mmol/L and free fatty acids 0.11 mmol/L. His Thyroid function tests were normal. Blood Autoantibody testing against insulin, *Tyrosine phosphatase-related islet antigen 2* (IA2) and *glutamic acid decarboxylase* (GAD) were all negative. Ultrasound revealed structurally normal pancreas and kidneys. On day 11 of life, normal term infant formula was changed to fully hydrolysed amino acid formula (*Neocate-LCP, Nutricia*) due to blood in stools, clinically consistent with cow’s milk protein colitis. There was a clinical response detected to this change with no further blood in stools.

From day 12 of life, the insulin infusion was gradually weaned and ceased by day 19 with stable BGLs of 4–6 mmol/L. Third hourly formula feeds (20 kcal/30 mL) via gastric tube were again established with regular BGL monitoring.

On day 44 of life, hypoglycemia (BGL 2.1 mmol/L) was detected on routine capillary pre-feed sugar monitoring. The infant at that point was on 170 ml/kg/day of 3^rd^ hourly formula tube feeds with an estimated glucose delivery rate (GDR) of 8.5 mg/kg/min and energy density of 20 kcal/30 mL. Further pre-feed glucose monitoring revealed recurrent asymptomatic hypoglycaemic episodes (BGL ranging 2.0–2.5 mmol/L). Critical blood sample collected at the time of hypoglycaemia (BGL 2.4 mmol/L) showed no evidence of hyperinsulinism, an appropriately suppressed insulin level (<2 mU/L), and detectable beta-hydroxybutyrate (0.19 mmol/L) levels. There was no response to Glucagon injection when tried to treat a random episode of hypoglycaemia (BGL 2 mmol/L), but resolved after increasing the energy density in the formula suggesting substrate deficiency as the cause. Of note, the baby’s weight standard deviation score had decreased from −2.17 at birth to −2.66. The energy density of the formula 20 kcal/30 mL was increased by adding glucose polymer (*Polyjoule, Nutricia*) (2 kcal/30 mL) to fortify it to 22 kcal/30 mL. This increased the glucose content of the milk to 8.7 % to give GDR of 10.3 mg/kg/min with 128 kcal/kg/day. The formula was then concentrated to 22 kcal/30 ml to make the total energy density 24 kcal/30 ml. This increased the glucose content to 9.6 % to give GDR to 11 mg/kg/min at 165 ml/kg/day with 130 kcal/kg and was required to maintain normoglycemia. The formula was concentrated to maintain the protein to energy ratio to allow adequate energy for growth secondary to IUGR. The increased fat and protein intake by concentrating the formula was intended to reduce the glycemic load rather than just add extra glucose. The carbohydrate in the Neocate-LCP is dried glucose syrup that has a high glycemic index.

The infant was discharged home on third hourly nasogastric tube feeds at 24 kcal/30 mL, at 170 ml/kg/day. Two oral feeds per day were allowed under the guidance of a speech pathologist. Parents were trained to perform pre-feed BGL at home and were educated to treat emergency hypoglycaemia at home with a glucose polymer solution (33 % concentration) to provide one hour’s worth of glucose administered as a bolus via nasogastric tube.

At 14 months of age, there was no further hypoglycaemia and his weight standard deviation score had improved to −0.32. The infant underwent a monitored fasting study for 12 h. At the end of the 12-h fast the BGL remained normal (3.3 mmol/L), with appropriately elevated beta-hydroxybutyrate (1.49 mmol/L) and free fatty acids (0.73 mmol/L). The following day the baby developed adenovirus gastroenteritis and was readmitted to hospital with hypoglycaemia, indicating a tendency to ketotic hypoglycaemia during intercurrent illness.

Genetic DNA testing for neonatal diabetes revealed no pathogenic mutation in common *KCNJ11, ABCC8* and *INS* genes. Mutations in these genes is the most common cause of permanent neonatal diabetes [1]. Methylation specific PCR of the infant’s DNA and microsatellite analysis on the infant and mother’s DNA at several markers within the TNDM critical region were performed. This revealed presence of partial hypo-methylation at the TNDM locus and increased paternal allele dosage in the patient, suggestive of paternal duplication of 6q24 locus. Subsequent analysis of paternal blood-derived DNA showed no evidence of duplication, suggesting that this duplication probably arose de-novo.

## Case discussion

Our infant presented with low birth weight and growth retardation along with the metabolic profile consistent with transient diabetes mellitus. The growth retardation is consistent with the finding with those with abnormality of the 6q24 gene locus for TNDM [1, 4] and is likely related to the failure of insulin secretion, which is a potent growth factor in fetal life [1].

Our infant, growth restricted at birth developed asymptomatic hypoglycaemia at 6 weeks of age with no evidence of hyperinsulism. His declining weight Standard deviation score and response to increased feed intake suggests substrate deficiency as the cause of his hypoglycaemic episodes. These hypoglycaemic episodes did not respond to glucagon treatment and diazoxide therapy was not tried as there was no evidence of hyperinsulism.

Asymptomatic hypoglycaemia as noted in our case is a very rare observation in this rare syndrome. We compare our case with the previous 6 cases reported by Flanagan et al. [4]. Flanagan’s report quoted 14 % incidence of hypoglycaemia in their cohort (6 out of 43 cases), all of which were symptomatic. The mean age of onset of symptomatic hypoglycaemia in their cases was 29 weeks. Five of their 6 cases had TNDM due to paternal uniparental disomy; of these 3 had hyperinsulinism (detectable insulin levels at the time of hypoglycaemia) and 2 had probable hyperinsulinism (undetectable insulin levels, but suppressed ketones at the time of hypoglycaemia, and zresponse to diazoxide therapy. The remaining case had a paternal duplication similar to our patient, but neither insulin nor ketones were measured at the time of hypoglycaemia. This case also had only short-lived episodes of hypoglycaemia with intercurrent illness (similar to our patient) whereas the other 5 had ongoing problems for 2 or more years (4 requiring diazoxide therapy and the other requiring continuous overnight feeds).

The genetic cause of transient neonatal diabetes mellitus has been attributed to overexpression of imprinted and paternally expressed gene/s within the TNDM critical region at 6q24 chromosome [4]. Two imprinted genes, *ZAC*(zinc finger protein associated with apoptosis and cell cycle arrest) and *HYMAI* (imprinted in hydatidiform mole) have been identified as potential candidates [1]. Three genetic mechanisms have been shown to result in TNDM: paternal uniparental isodisomy of chromosome 6, paternally inherited duplication of 6q24, and a methylation defect at a CpG island overlapping exon 1of *PLAGL1*/*HYMAI* [4]. Our case was detected to have a partial hypomethylation at the TNDM locus of 6q24 and also had additional paternal allele dosage detected on the 6q24 gene. Thus, in view of the absence of a significant methylation defect, the abnormality in our patient was attributed to the duplication of paternal 6q24 gene, resulting in TNDM.

With regards to timing of BGL monitoring, hypoglycemia in our infant was detected on pre-feed sugar monitoring while on 170 ml/kg/day of 3rd hourly enteral feeds. Post-feed BGL following these hypoglycaemic episodes were always normal. Pre-feed detection of low BGL resulted in early detection of hypoglycemia. There is no study that looks specifically at the optimal timing and intervals for glucose screening. Our case demonstrates that the brief period of asymptomatic recurrent hypoglycaemia would have been likely missed, if only post feed BGLs were monitored.

Animal model studies have shown an increase in the number of pancreatic beta cells prior to diabetes remission and it is possible that in some patients there is an ‘overshoot’ of this process and consequently beta cell hyperplasia [5], however our patient had undetectable insulin during the episode of hypoglycemia.

## Conclusion

Thus, it is important to be aware of the risk of hypoglycemia in early days of life following remission of diabetes mellitus in patients with 6q24 chromosome defect. We suggest regular pre-feed BGL monitoring in these infants to identify subclinical hypoglycaemia which may not be noticed on post-feed BGL monitoring alone.

## Patient Consent

Written informed consent was obtained from the parent (mother) of the patient for publication of this case report. A copy of the written consent is available for review by the Editor of this Journal. The present case report was performed in accordance with the institutional ethics regulations.

## Abbreviations

BGL: Blood glucose level
GDR: Glucose delivery rate
IUGR: Intrauterine growth retardation
TNDM: Transient Neonatal diabetes Mellitus
